# Medicinal Properties and In Vitro Biological Activities of Selected Helichrysum Species from South Africa: A Review

**DOI:** 10.3390/plants10081566

**Published:** 2021-07-30

**Authors:** Kolajo Adedamola Akinyede, Christopher Nelson Cupido, Gail Denise Hughes, Oluwafemi Omoniyi Oguntibeju, Okobi Eko Ekpo

**Affiliations:** 1 Department of Medical Bioscience, University of the Western Cape, Private Bag X17, Bellville 7530, South Africa; ghughes@uwc.ac.za; 2 Department of Science Technology, Biochemistry Unit, The Federal Polytechnic P.M.B.5351, Ado Ekiti 360231, Nigeria; 3 Department of Botany, University of Fort Hare, Private Bag X1314, Alice 5700, South Africa; ccupido@ufh.ac.za; 4 Phytomedicine and Phytochemistry Group, Oxidative Stress Research Centre, Department of Biomedical Sciences, Faculty of Health and Wellness Sciences, Cape Peninsula University of Technology, P.O. Box 1906, Bellville 7535, South Africa; oguntibejuo@cput.ac.za; 5 Department of Anatomy and Cellular Biology, College of Medicine and Health Sciences, Khalifa University, Abu Dhabi P.O. Box 127788, United Arab Emirates

**Keywords:** medicinal plants, phytochemicals, pharmacological actions, *Helichrysum* species

## Abstract

The genus *Helichrysum* Mill comprises hundreds of species that are mostly flowering perennial shrubs. Some of these plants that belong to the *Helichrysum* species are used in traditional medicine to treat cough, back pain, diabetes, asthma, digestive problems, menstrual pain, chest pain, kidney disorders, skin disorders, wounds, open sores, among other conditions, but, only a few scientific studies are reported in the literature with sufficient information that validates the acclaimed folkloric benefits of these plants. This review, therefore, provides a comprehensive update of the available information on the cytotoxicity, genotoxicity, anti-proliferative, anti-bacterial, anti-fungal, anti-viral, anti-HIV, anti-malarial, anti-ulcerogenic, anti-tyrosinase, anti-inflammatory, and anti-oxidant activities of selected *Helichrysum* species of interest: *H. petiolare*, *H. cymocum*, *H. foetidum*, and *H. pandurifolium* Schrank, using scientific databases as well as electronic and print sources. The ethnobotanical and morphological characteristics as well as the phytochemical composition and biological activities of these plants are elucidated. The scientific rationale for their current use is discussed based on the evidence in the literature. This review highlights the putative use of the *Helichrysum* species as a reliable source of bioactive compounds for the production of standard commercial drugs to treat many ailments, including those reported in folkloric uses. Further research on the many plants in the genus *Helichrysum* is recommended to explore their economic importance both as edible crops and medicinal botanicals.

## 1. Introduction

The genus *Helichrysum* Mill comprises distinctively of aromatic herbs and shrubs of the family *Asteraceae*. It has a worldwide distribution but is mainly found in Africa, with its highest diversity in South Africa, where approximately 245 of the 500 known species occur. Other areas of *Helichrysum* diversity include Europe, southwestern Asia, South India, Sri Lanka, Turkey, and Australia. For ease of identification, the southern African species are divided into 30 informal groups [1].

Plants of the *Helichrysum* genus have been in use for more than 2000 years for various folkloric purposes. The flowers of some members of this genus have a unique bright-yellow color that depicts their Greek language origins: “helios” and “chryos” which mean “sun” and “gold” respectively. In folkloric medicine, some *Helichrysum* plant parts are either consumed as teas or prepared as “burnt offering” smoke to disinfect the abodes of sick patients and to appeal for blessings from the ancestors in indigenous traditional practices [2]. In general, plants of this genus are known to be used in traditional medicine for the treatment of many ailments, including liver disorders, gall bladder complications, cystitis, jaundice, stomach pain, allergies, infections, colds, cough, skin infections, inflammation, menstrual pain, asthma, arthritis, insomnia, diabetes mellitus, and for wound healing [3,4,5,6,7], most commonly, the scented leaves and flowers.

The therapeutic properties of the *Helichrysum* species are often attributed to their different constituent phytochemicals, especially the essential oils [8,9]. In addition to the essential oils, plants in this species also contain such phytochemicals as terpenoids, phenolics and oxygenated compounds as secondary metabolites, including flavonoids, chalcones, phenolic acids, terpenes and essential oils, pyrone, benzofurans, and phloroglucinols [10]. Thus, these plants are potential reservoirs of bioactive compounds for drug discovery and development. Hitherto, only limited biological effects of the *Helichrysum* phytochemicals have been reported including, the antioxidant, antifungal, anti-inflammatory, anti-bacterial, hepatoprotective, anti-proliferative and anti-diabetic activities [7,11,12,13].

So far, only a few of the many *Helichrysum* species have been studied; the best known *Helichrysum* species used traditionally to treat different ailments are *H. cymosum*, *H. odoratissimum*, *H. petiolare* and *H. nudifolium*. Only limited information is available in the literature on these plant species on their ornamental, industrial and pharmaceutical applications [14]. Thus, in this review, peer-reviewed information on *Helichrysum petiolare* Hilliard & B. L Burtt, *Helichrysum cymosum* (L.) D.Don, *Helichrysum foetidum* (L.). Moench and *Helichrysum pandurifolium* Schrank from South Africa will be elaborated, including their botanical, ethnopharmacological, phytochemical and bio-scientific profiles.

## 2. Research Methodology

The keywords relevant to this review, including “*Helichrysum species*” “*Helichrysum petiolare* Hilliard & B. L Burtt”, “*Helichrysum cymosum* (L.) D. Don”, “*Helichrysum foetidum* (L). Moench”, “*Helichrysum pandurifolium* Schrank”, “cytotoxicity”, “anti-genotoxicity”, “anti-proliferative”, “anti-bacterial”, “anti-fungal”, “anti-viral”, “anti-malarial”, “anti-ulcerogenic”, “anti-tyrosinase”, “anti-inflammatory”, “antioxidant”, “phytochemicals”, “ethnopharmacology” and “essential oil” were searched for, using different databases including Sci Finder, ISI Web of Knowledge, Science Direct, Google Scholar, PubMed, Scopus, Wiley Online Library and Springer; while online theses, dissertations, and other print materials also provided important information on the traditional use, bioactive phytochemical compositions, biological and pharmacological actions, as well as leads on relevant gaps in research and future directions.

## 3. *Helichrysum petiolare* and Its Biological Activities

*Helichrysum petiolare* (*H. petiolare*) is commonly referred to as “Silverbush everlasting plant”, and called “kooigoed” in the Afrikaans language of South Africa. This plant is a shrub with gray or silver-gray hair covering the aromatic round-shaped leaf, while its flowers are whitish-creamy [15]. *H. petiolare* is used in South African traditional medicine to treat fever, catarrh, cold, cough, menstrual disorders, kidney-related infections, chest problems, high blood pressure, and erectile dysfunction. In addition, the decoction of the leaves of the plant is used in traditional beauty therapy to refine skin texture/looks and for wound healing [16]. The known phytochemicals present in *H. petiolare* include phenols, flavonoids, and anthocyanins, the extract and its essential oil constituents were reported to show activity against Gram-positive and Gram-negative bacteria [17]. Other biological activities of this plant are discussed below.

### 3.1. Cytotoxicity/Anti-Proliferative Activity of H. petiolare

The in vitro cytotoxic effects of the chloroform and methanol leaf and stem extracts of *H. petiolare* (1:1 solvent ratio) on transformed kidney epithelial (Graham’s) cells, breast cancer cells (MCF-7) and brain cancer (SF-268) cells were studied using the sulforhodamine B (SRB) assay and the results showed percentage growth inhibition of 59%, 33% and 76% for the Graham’s, MCF-7, and SF-268 cell lines, respectively [18]. In another study, B16F10 mouse melanoma cells and MeWo human skin melanoma cells were treated with the methanol extract of *H. petiolare*, and the cytotoxicity and cell cycle analysis showed a dose-dependent reduction in cell viability or proliferation, as well as S-phase and M-phase cell cycle arrest, reflecting pro-apoptotic effects. 

### 3.2. Anti-Bacterial Activity of H. petiolare

The anti-bacterial activities of nine *Helichrysum* species of South African origin, including *H. petiolare*, have been reported in such bacterial strains as *Escherichia coli*, *Yersinia enterocolytica*, *Klebsiella pneumomiae* (Gram-positive) and *Staphylococcus aureus* and *Bacillus cereus* (Gram-negative), using disc diffusion assay, with the standard drug neomycin as control [19]. When compared with the control, the methanol and acetone extracts of *H. petiolare* showed significant activities against *S. aureus* and *B. cereus* strains while the essential oils showed no activity against these two bacterial strains. The zone of inhibition is a uniformly circular area on an antibiotic dish that has no bacterial growth [20]. In the study under reference, the inhibition zone values ranged between 2.5 mm to 9.0 mm in the methanol and acetone extract-treated dishes compared with the 6 mm zone of inhibition in the standard drug. The Gram-positive bacteria were unaffected following treatment with the essential oil as well as the methanol and acetone extract of *H. petiolare*. Furthermore, the minimum inhibitory concentration (MIC) assay was used as a confirmatory test for the disc diffusion assay, and the results of this assay showed much improved activity for both the methanol and acetone extracts (<0.25 mg/mL) of *H. petiolare* when compared with the standard drug ciprofloxacin (0.31 × 10^−3^ mg/mL). However, the MIC assay showed a relatively low activity of the essential oil treatment (8 mg/mL) on the *S. aureus* bacterial strain [19].

In another study, Lourens and co-workers reported on the anti-bacterial effects of the methanol and chloroform (1:1 solvent ratio) extracts of *H. petiolare* on five bacterial strains, namely *B. cereus*, *S. aureus*, *S. epidermidis*, (Gram-positive), *K. pneumomiae*, and *P. aeruginosa* (Gram-negative), with ciprofloxacin as the positive control. The results showed minimum anti-bacterial effects against *S. aureus* and *B. cereus* with a MIC value of 4 mg/mL and 2 mg/mL respectively, compared to the control. However, no anti-bacterial activity was recorded against the remaining bacterial strains in the 96-well microplate assay [21].

### 3.3. Anti-Inflammatory Activity of H. petiolare

Louren and co-workers investigated the anti-inflammatory properties of the essential oil of *H. petiolare* using the 5-lipoxygenase assay, and the half-maximal inhibitory concentration (IC_50_) value of 23.05 ± 0.57 µg/mL was obtained for the varying concentrations of hydro-distilled essential oils, indicating potent anti-inflammatory effects. In comparison, the positive control had an IC_50_ value of 5.0 ± 0.50 µg/mL while the methanol and acetone extract of *H. petiolare* showed no anti-inflammatory activity, with an IC_50_ value greater than 100 µg/mL [21].

### 3.4. Anti-Fungal Activity of H. petiolare

Three strains of human fungi namely, *Cryptococcus neoformanas*, *Candida albicans* and *Alternaria alternate*, were evaluated using the disc diffusion assay. The results showed that only the acetone extract of *H. pertiolare* showed moderate anti-fungal activity against *C. albican* compared with the standard drug Nystatin. In contrast, the essential oil and methanol extract had no anti-fungal activity in the three fungal strains tested [21].

### 3.5. Anti-Oxidant Activity of H. petiolare

A slight modification of the 1,1 diphenyl -2- picrylhydrazyl (DPPH) free radical scavenging assay [20] was used to determine the anti-oxidant activity of the extracts of *H. petiolare*, in reference to the ascorbic acid standard. The results of treatment with the IC_50_ values of 44.28 µg/mL, 28.70 µg/mL and 2.5 µg/mL of the acetone and methanol extracts as well as the ascorbic acid standard, respectively, showed that more than 50% of the free radicals was inhibited or neutralized.

### 3.6. Antigenotoxicity Activity of H. petiolare

The results from one study showed genotoxic effects of the methanol extracts of *H. petiolare* on normal Vero cells at concentration ranges of 12.5–200 µg/mL, using the micronucleus assay [22]. In another study, the 90% methanol and dichloromethane (DCM) extracts of *H. petiolare* were screened for aflatoxin B_1_-induced mutagenicity using the bacteria-based genotoxic Ames and Vitotox assays [23]. The methanol extract showed significant dose-dependent anti-mutagenic activity against *S. typhimurium* TA100 and TA 98 while the DCM extract had moderate anti-mutagenic effects against *S. typhimurium* TA100 and more severe effects against *S. typhimurium* TA98. Cancer remains a global disease today with high mortality and morbidity rate. Mutagens are known to promote the pathogenesis of most cancers via gene mutation and chromosomal aberration and anti-mutagens are known to prevent chromosomal translocation, deletion and inversion [22,23]; hence the use of *H. petiolare* extracts as food supplements could serve as potential carcinoprotection agents, especially cancers induced by aflatoxin-producing fungi, in poorly-processed food crops.

### 3.7. Anti-Tyrosinase Activity of H. petiolare

A number of skin disorders are known to be promoted by anomalies in such implicated enzymes as tyrosinase and elastase, in the face of damaging free radical effects on the skin [24]. Therefore, compounds that inhibit such enzymes (e.g., tyrosinase inhibitors) could potentially be very useful in the cosmetic industry to prevent skin aging and other undesirable skin conditions [25,26]. *H. petiolare* plant extracts have been investigated for their tyrosinase inhibition activities (25 ), and results obtained showed moderate tyrosinase inhibition values of 44.3% and 59.2% at 50 µg/mL and 200 µg/mL concentrations respectively, using kojic acid as the reference control [25]. The results obtained tend to suggest that phytochemicals from the extracts of *H. petiolare* could inhibit free radical accumulation and modulate tyrosinase activity. 

In another study, treatment with the ethanol extract of *H. petiolare* was found to be non-toxic to human dermal fibroblast (MRHF) cells using the Hoechst 3342/propidium iodide stain and resulted in decreased ROS and NO production following lipopolysaccharide-induced damage in the RAW 246.7 cell line. Additionally, weak inhibitory effects of the extract on the enzymes of collagenase, elastase and tyrosinase at various concentrations was reported in another study, compared to the positive control. In addition, this plant extract effectively inhibited protein glycation, indicating its potential use as an anti-aging agent for the skin [27].

## 4. *Helichrysum cymosum* and Its Biological Activities

*Helichrysum cymosum* (*H. cymosum*) is a plant endemic to South Africa and known commonly as “gold carpet” or “yellow-tipped strawflower” in English. Its local South African names include “goue tapyt” (in Afrikaans) and “impehho” (in isiXhosa). This plant is widely distributed in the Eastern Cape, KwaZulu-Natal, and Western Cape Provinces, and grows as a short, wooly shrub with grayish silver leaves and bright yellow-colored flowers in flat heads. This species is divided into two subspecies, namely the *H. cymosum* subsp. *cymosum* and *H. cymosum* subsp. *calvum* Hilliard [28,29], with distinct characteristics in the flowers, fimbrils, ovary, pappus, and plant length [29]. Traditionally, the aerial parts, leaves, roots, and leaves of this plant are used to treat different ailments ranging from cough, catarrh, colds, headache, menstrual pain, fever, wounds, flatulence, pulmonary problems, skin infections, pertussis vomiting as well as to improve immunity, and appetite [15,29]. The reported biological and pharmacological activities of *H. cymosum* include anti-bacterial, anti-fungal, anti-inflammatory, anti-malarial, anti-oxidant, and cytotoxic effects [15,29], some of which are briefly discussed below.

### 4.1. Cytotoxicity of H. cymosum

Vuuren et al. investigated the toxicity of the essential oil and acetone extract of *H. cymosum* as well as one of its isolated compounds, helihumulone on the transformed kidney epithelial cell line, using the tetrazolium-based cell proliferative or viability assay (MTT) [30]. The results showed that the essential oil was the most toxic extract, with an IC_50_ value of 17.47 ± 33.0 μg/mL compared to 172.2 ± 10.08 μg/mL for the acetone extract and 57.05 ± 3.04 μg/mL for helihumulone, respectively. In another study, the cytotoxic effects of *H. cymocum* on the kidney Vero cell line were evaluated using the XTT assay with varying concentrations of different solvent extracts of the plant: 3.13, 6.25, 12.50, 25.00, 50.00, 100.00, 200.00 and 400.00 µg/mL. These results showed that compared to the positive control Zearlenone, with an IC_50_ value of 1.33 ± 0.32 µg/mL, the chloroform extract (IC_50_ value 36.52 ± 0.27 µg/mL) was more toxic to the Vero cells than the methanol-water (50% water/methanol and 50% chloroform) extract with an IC_50_ value 59.74 ± 0.14 µg/mL [31].

### 4.2. Anti-Oxidant Activity of H. cymosum

Francois (2016) investigated the anti-oxidant capacity of the essential oils isolated from *H. cymosum* leaves using the 1, 1 diphenyl 1- picrylhydrazyl (DPPH) assay. Results obtained showed that 50% free radical scavenging activity was achieved by the 6.3 g/L concentration of this plant compared to the same effects by the 7.0 mg/L concentration of the positive control, butylated hydroxytoluene (BHT), a known compound used as a food anti-oxidant additive [29]. Thus, the extract of this plant had more anti-oxidant effects than BHT, indicating potential use as food additives. No other anti-oxidant studies involving *H. cymosum* were found in the literature at the time of writing this review, hence more studies are recommended.

### 4.3. Anti-Malarial Activity of H. cymocum

Vuuren et al. reported on the anti-plasmodium activity of the acetone crude extract, the essential oil, and the isolated compound helihumulone from *H. cymocum* on the falciparum parasite, relative to the effects of two standard anti-malaria drugs (quinine and chloroquine). The results from the G3-H hypoxanthine incorporation assay showed that when compared to the two standard anti-malarial drugs, quinine (IC_50_ value 0.13 ± 0.04 μg/mL) and chloroquine (IC_50_ value 0.09 ± 0.02 μg/mL), the essential oils had the least IC_50_ value (1.25 ± 0.77 μg/mL) followed by helihumulone (14.89 ± 1.88 μg/mL) and then the acetone crude extract (60.76 ± 2.83 μg/mL). Considering the relatively low IC_50_ value of the essential oils of *H. cymocum*, their potential use as natural sources of anti-malarial treatment agents is plausible [30]. 

### 4.4. Anti-Fungal Activity of H. cymocum

The anti-fungal activity of the essential oil and acetone extract of *H. cymocum* as well as the isolated compound, helihumulone against *C. neoformans* and *C. albicans* was investigated using the microdilution technique, with a potent anti-fungal agent amphotericin B as the positive control. The MIC values used ranged from 0.03–4.0 mg/mL, and the results showed that the 0.03 mg/mL and 0.063 mg/mL MICs of the helihumulone were the most potent against *C. neoformans* and *C. albicans* [32]. Another study on the anti-fungal activity of the isolated essential oils of *H. cymocum* showed that over 50% zone of inhibition was achieved against *Penicillium oxalicum* while a 6 mm to 9 mm area of inhibition was reported for *C. albicans* [33,34].

### 4.5. Anti-Bacterial Activity of H. cymocum

Bougatsosa et al. reported on the anti-bacterial activities of the essential oils of *H. cymocum* against the Gram-positive bacteria *S. aureus* and *S. epidermis* as well as four Gram-negative bacteria, *E. coli*, *Enterobacter cloacae*, *K. pneumoniae* and *Pseudomonas aeruginosa*. The MIC results from the dilution technique showed that the essential oils of *H. cymocum* were not sensitive (completely inactive) to all the bacteria strains tested when compared to the essential oils of a closely-related plant species *H. fulgidum* (L.) Wild, which have been reported to be highly sensitive to the same bacterial strains tested [34]. Similar studies by Sindambiwe et al. showed that the 80% ethanol extract of *H. cymocum* was not sensitive to the bacteria *Proteus vulgaris*, *E. coli*, *K. pneumoniae*, *P. aeruginosa*, *Salmonella paratyphi*, *B. cereus*, *Mycobacterium fortuitum* and *S. aureus*, whereas *Streptococcus pyogenes* showed a MIC value of 5 mg/mL which indicates some sensitivity [35]. The results of another anti-bacterial activity study using the disc diffusion assay showed a zone of inhibition value of 7 mm and 5 mm respectively for *S. aureus*, 8 mm and 5 mm respectively for *B. cereus*, and no inhibition by the essential oils [32]. In yet another study, the isolated compound helihumulone, was found to have the highest anti-bacterial activity against the bacteria *Enterococcus faecalis*, *B. cereus*, *B. subtilis*, *S. aureus*, *P. aeruginosa*, *E. coli*, *Y. enterocolitica*, and *K. pneumonia* at the concentration range of 0.02–0.12 mg/mL, using the p-iodo nitro tetrazolium (INT) microplate method, when compared with the essential oil (1.0–8.0 mg/mL) and acetone extract (0.1–0.3 mg/mL) of *H. cymocum*. The acetone extract activity was reported to be six times more than that of the essential oils [33].

### 4.6. Anti-Inflammatory Activity of H. cymocum

Standford and co-workers investigated the inhibitory effects of the ethanol and aqueous extracts of *H. cymocum* to prostaglandin, a known marker of inflammation, using the in vitro cyclooxygenase assay. The results showed that, whereas the standard anti-inflammatory drug indomethacin showed 60% inhibition, the aqueous extract showed 52% and the ethanol extract showed 100% inhibitory activity [36]. In addition, other related plant species like *Helichrysum excisum* and *Helichrysum felinum* were reported to exhibit anti-inflammatory properties; the acetone extract and essential oil from *H. excisum* showed anti-inflammatory properties at IC_50_ concentrations of 35.09 ± 1.12 µg/mL and 27.62 ± 0.43 µg/mL respectively, using the 5-lipoxygenase assay, while the anti-inflammatory properties reported for the acetone extract and essential oil from *H. felinum*, occurred at IC_50_ concentrations of 38.72 ± 2.94 µg/mL and 22.87 ± 7.59 µg/mL respectively, in comparison with control with IC_50_ of 5.00 ± 0.50 µg/mL [37].

### 4.7. Anti-Viral Activity of H. cymocum

The virucidal activity of the ethanol extract of *H. cymocum* against selected viruses was studied using the 50% endpoint titration technique (EPTT). This assay involves the determination of virus titer reduction in the presence of two-fold dilutions of test compounds on monolayers of cells grown in plastic or glass Petri dishes [38]. The results obtained showed that the extract was effective against the simplex virus type 1 (HSV1), the measles virus strain Edmonston A (MV-EA) as well as the Semliki forest virus A_7_ (S.F. A_7_) [35]. In addition, the anti-viral bioactivity of the methanol/water and chloroform (50% water/methanol and 50% chloroform) extracts of the aerial parts of *H. cymocum* were investigated in one study using colorimetric cell-based, a cytopathic effect inhibition assay. The results showed cytopathic effects against Vero cells at a final toxic concentration of 400 μg/mL, compared with the positive control acyclovir with anti-viral activity of 0.75 μg/mL [31].

## 5. *Helichrysum foetidum* and Its Biological Activities

*H. foetidum* is a robust herb commonly referred to as the stinging starflower with a characteristic pungent smell. Its leaves are elliptic, sparsely hairy, white-woolly, and are arranged in groups at the base of the stem while its many flower heads are borne in broad, leafy spreading umbrella-like inflorescences [39]. This plant is native to South Africa and has since been introduced to other parts of the world, including Spain, Portugal, and Southern Brazil [40]. *H. foetidum* is used in African tradomedical practices to induce a trance, mainly due to its hallucinatory effects. It is also used for the treatment of wounds, for dressing the circumcised penis in traditional ceremonies, for infected sores, for menstrual pains, herpes, eye infection, influenza, among other ailments [39]. The chemical composition of *H. foetidum* includes diterpenoid, Kaur-16-en-18-oic acid, flavonoid, apigenin 7,4 O-dihydroxy-5-methoxy-flavanone, apigenin 7-O- β-D- glucoside and 6-methoxy-2′, 4,4-trihydroxy-chalcone helichrysetins, as well as glucosylated helichrysins [41]. Its biological activities include anti-bacterial [42], anti-fungal [43], anti-viral [35], antioxidant, anti-ulcerogenic, and cytotoxic effects [43].

### 5.1. Cytotoxicity of H. foetidum

The in vitro cytotoxic effects of the isolated compounds of *H. foetidum* on the human prostate cancer cell line (PC-3) were evaluated using the XTT assay. These compounds were found to have no effects on PC-3 viability at the concentrations. It was reported that the compound reduced the viability of cancer cells and induced apoptosis at the concentrations of 50 nM or 50 μM of the isolated compound [42]. In another study, the transformed human kidney epithelial (Graham’s) cells, MCF-7 breast adenocarcinoma cells, and SF-268 glioblastoma cells were reported to be sensitive to the leaf and stem extracts of chloroform and methanol (solvent ratio 1:1), with a significant reduction in cell viability (24.9%) in MCF-7 cells, at a concentration of 0.1 mg/mL of the extract [18].

### 5.2. Anti-Ulcerogenic Properties of H. foetidum

Malolo et al. reported on the potential of the methanol extract and the isolated compounds from *H. foetidum*, to inhibit the enzyme protease, through the in vitro protease pepsin inhibition assay known as the fluorescence resonance energy transfer (FRET) assay [43]. Pepsin is the main acid protease of the stomach and is implicated in peptic ulcer disease, reflux oesophagitis, and excessive stomach acid secretion, although *Helicobacter pylori* bacteria are also involved in the pathogenesis of most gastric diseases. Following treatment with the methanol extract of *H. foetidum*, the FRET assay showed 17.8%, 35.6% and 37.6% inhibitory activity against pepsin at the concentrations of 10 μg/mL, 25 μg/mL and 50 μg/mL, respectively; however, no action was detected against subtilisin, a known alkaline protease [43,44]. Furthermore, out of the six isolated compounds tested in this study, only apigenin-7-β-D-glucoside and 6′ methoxy-2′,4 dihydroxychalcone-4′-*O*-β-D-glucoside showed moderate inhibition range of 37.4% to 46.3% at 50 μg/mL concentration [43]. 

### 5.3. Anti-Bacterial Activity of H. foetidum

Steenkamp et al. (2014) investigated the anti-bacterial activity of the methanol, and aqueous extracts of *H. foetidum* against *S. aureus*, *S. pyrogenes*, *E. coli*, and *P. aeruginosa*, and the results showed a MIC value of not more than 4 mg/mL in all the bacterial strains tested [45]. In addition, the anti-bacterial activity of the methanol extract and six isolated compounds from *H. foetidum* against *B. subtilis* bacteria was evaluated in another study, using a fluorescence-based anti-bacterial inhibition assay [43]. The results obtained showed MIC values of 85.4% and 21.8% for the methanol extract, at the concentration range of 1.0 mg/mL and 0.1 mg/mL respectively, while the isolated compounds displayed growth inhibition in the range of 75.0% to 85.0%.

### 5.4. Anti-Fungal Activity of H. foetidum

*Cladosporium cucumerrinum* is a known fungus that causes diseases in plants, leading to reduced crop yield. The in vitro anti-fungal activity of the methanol extract and six isolated compounds from *H. foetidum* against this pathogen was reported [45]. The concentrations of 50 μg/cm, 100 μg/cm, 200 μg/cm and 400 μg/cm of the methanol extract of *Helichrysum foetidum* used in a bioautograpy assay on silica gel plates, indicated that zones of growth inhibition against the fungus were well developed on bioautograpy plate, showing a significant anti-fungal property [45]. All the six isolated compounds showed growth inhibition against *C. cucumerrinum* in the range of 70% to 56% at 1.0 mg/mL concentrations in the assay [45].

### 5.5. Anti-Oxidant Activity of H. foetidum

Bruno et al. reported the anti-oxidant activity of the methanol extract of *H. foetidum* using an array of in vitro assays [46], including the 2-2’ azinobin-3-ethlbenzothiazoline-6- sulphonic acid (ABTS), 1,1-diphenyl-2-picrylhydrazyl (DPPH) radical-scavenging, and the β-carotene/linoleic acid assay. The anti-oxidant activity of the methanol extract of *H. foetidum* determined by the ABTS and DPPH assays, occurred at the IC_50_ concentrations of 0.5 ± 0.1 μg, 0.6 ± 0.1 μg, and 1.5 ± 0.3 μg respectively, compared with the known anti-oxidant compounds, Trolox or BHT, used as the standards, with IC_50_ values of 1.3 μg and 1.5 μg respectively. On the other hand, the scavenging of hydrogen peroxide (HRPO) test, the superoxide anion scavenging (SAS) test and the hypochlorous acid scavenging (taurine) test showed anti-oxidant activity at IC_50_ concentrations of 15.0 ± 2.0 μg, 34.0 ± 6.0 μg, and 24.0 ± 3.0 μg respectively. The anti-oxidant activity of medicinal plants is commonly attributed to the presence of phenols, flavonoids, flavanols, and oligomeric proanthocyanidins, which in this study, were quantified to be 580 ± 87 mg/g, 460 ± 69 mg/g, 12 ± 2 mg/g, and 5 ± 1 mg/g respectively, in the methanol extract of *H. foetidum* [46].

### 5.6. Anti-Viral Activities of H. foetidum

The 50% endpoint titration technique (50% EPTT) was adopted by Sindambiwe et al. (1999) to evaluate the anti-viral activities of the aqueous and 80% ethanol extracts of the whole plant parts of *H. foetidum*. Virucidal activities against HSV1 and the Semliki Forest virus A7 (SFA7) were reported but not against the measles virus strain Edmonston A (MV-EA) and the vesicular stomatitis virus T2 (VSVT2) [35].

## 6. *Helichrysum pandurifolium* Schrank and Its Biological Activities

The common name of *H. pandurifolium* Schrank is *Hottentotskruie* or *Hottentotskooigoed* or fiddle leaf-strawflower, with the stem and leaf parts often prepared as infusions for the traditional treatment of such ailments as respiratory conditions, cough, heart conditions, kidney stones and other kidney-related conditions [10]. The plant is a tall, slender, loosely-branched and soft shrub with orbicular-to-ovate leaves that abruptly narrow at a broad petiole-like base. The flowers are yellow, with the outer and inner parts of the involucre bracts being light-brown and white-pink, respectively. The apex of this herbal plant is sharp, while its fruits have a pappus with an array of feathery bristles [14,40].

Currently, information on the phytochemicals and pharmacological actions of *H. pandurifolium Schrank* is scanty in the literature, a possible indication that this species is not well-studied scientifically.

## 7. Common Phytochemicals Present in the Selected *Helichrysum* Species

**Figure 1:**
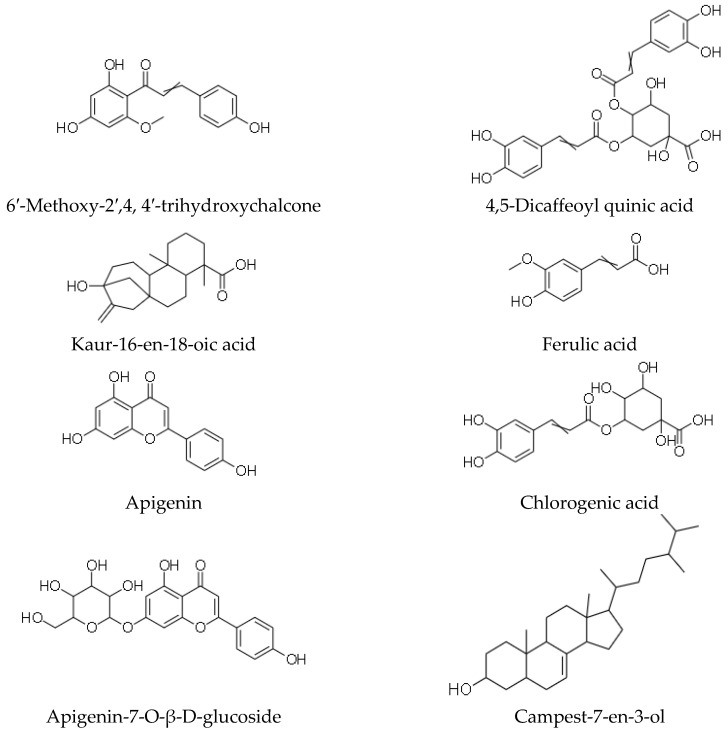

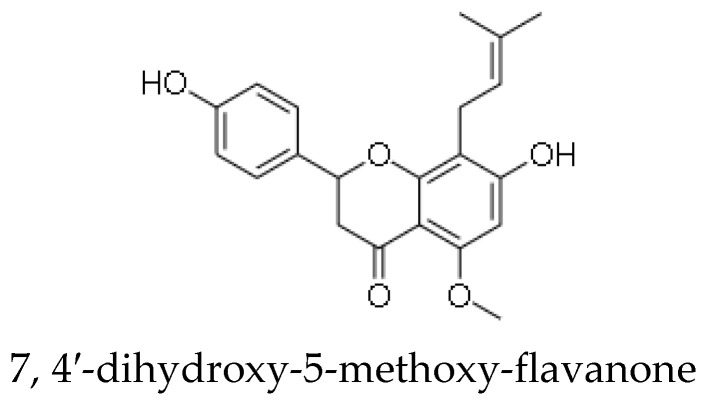
Structures of phytochemicals isolated from plants of *Helichrysum foetidum*.

**Figure 2:**
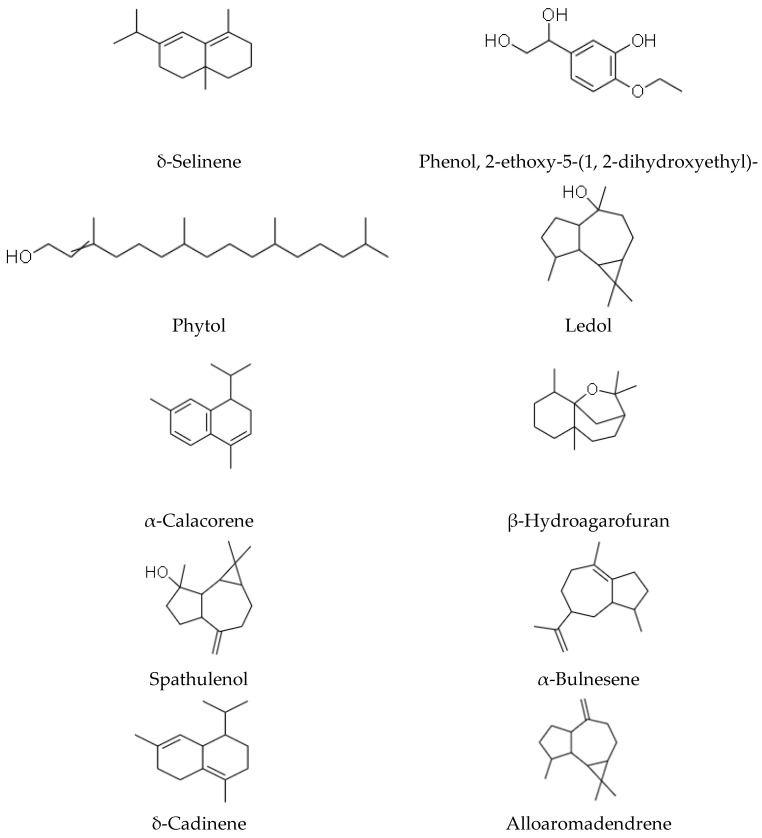
Structures of phytochemicals isolated from *Helichrysum petiolare*.

**Figure 3:**
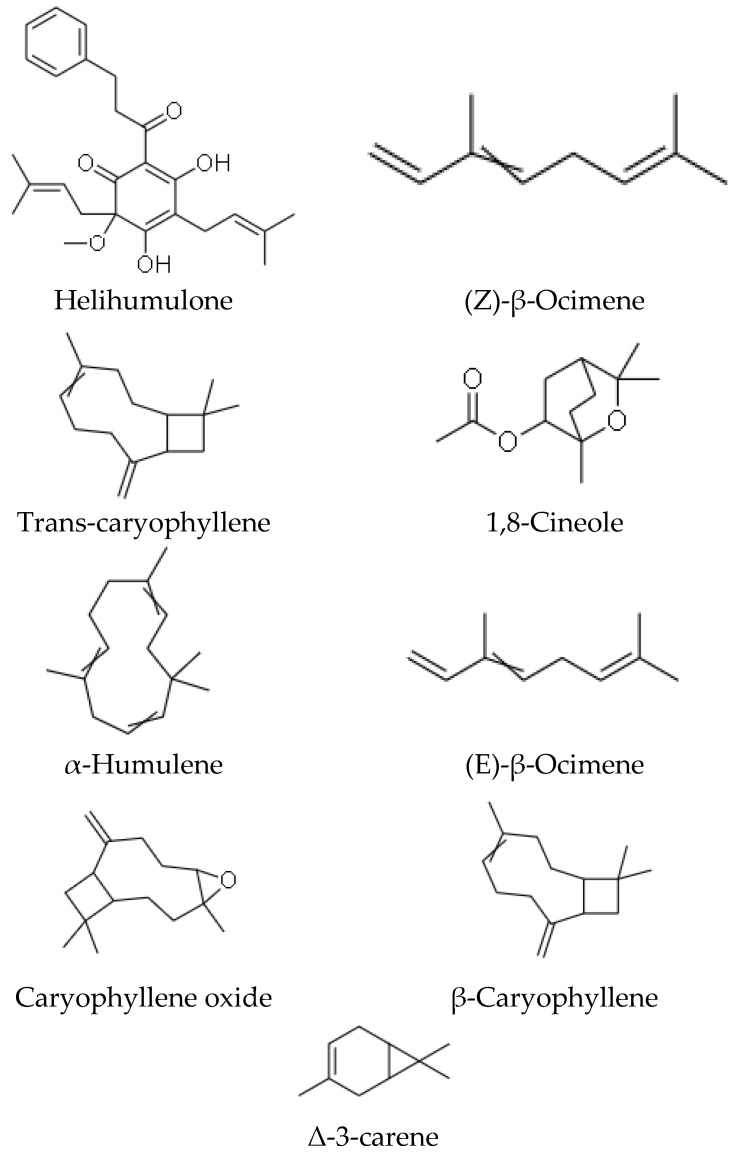
Structures of phytochemicals isolated from *Helichrysum cymosum*.

**Figure 4:**
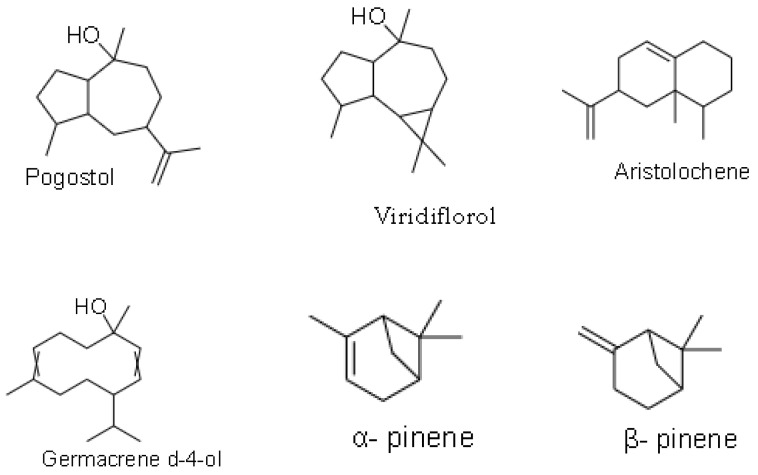

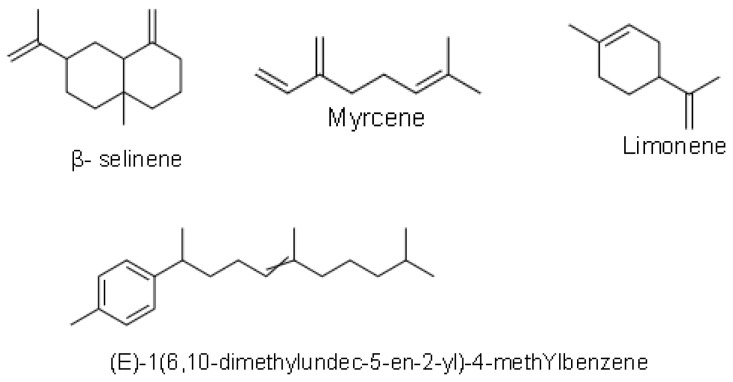
Structures of phytochemicals isolated from *H. pandurifolium*.

Phytochemicals refer to structurally diverse secondary metabolites or compounds that are produced by plants or non-pathogenic endophytic microorganisms in plants, to serve a protective role against any form of insults by pathogens (bacteria, fungi and viruses), through participation in the body’s anti-oxidant defensive and protective mechanisms such as free-radical scavenging [47,48]. Phytochemicals, therefore, confer essential pharmacological or biological functions on medicinal plants. In 1967, phytochemical studies of the *helichrysum* genus were done, and the helichrysum *dendroideum* species was the first to be explored [49], leading to the identification of many chemical secondary metabolites. Scientific reports on 63 *helichrysum* species of South African origin have shown many isolated compounds, including acylphloroglucinol, humulone derivatives, flavonoids, 8-hyroxyflavonols, α-pyrones, chalcone, and pyranochalcones [37,50,51]; essential oils, benzofurans, oxygenated compounds are present in the many species of this plant genus [10]. Representative of some of the compounds present in *H. petiolare*, *H. cymosum*, *H. foetidum* and *H. pandurifolium Schrank* are depicted in Figure 1, Figure 2, Figure 3 and Figure 4. While the biological functions of the associated compounds including that of the essential oils are listed in Table 1, Table 2, Table 3, Table 4 and Table 5 in this review.

## 8. Essential Oils Present in Selected *Helichrysum* Species

Essential oils (EOs) are the condensed or concentrated hydrophobic liquids that contain volatile chemical compounds derived from plants. The variety of compounds in EOs depends on such factors as the plant species, plant part used (leaves, fruits, roots), harvest period, environmental conditions (land fertility, humidity, temperature) and the extraction technique employed [68].

One distinct characteristic of the *helichrysum* genus is its aromatic nature and most of the species in this genus have been reported to be rich in essential oils (EOs) containing many compounds [10]. Many EOs are prescribed as alternative medicine (in aromatherapy) based on the healing effects of their aromatic compounds [69]. Monoterpenes, sesquiterpenes and diterpenes are some of the broad groups of compounds present in the EOs of most plants, including the *helichrysum* genus; these compounds are largely responsible for the reported anti-fungal, anti-bacterial, anti-diabetic, anti-inflammatory, anti-ulcer, anti-cancer, anti-oxidant, anti-nociceptive, and anti-spasmodic properties associated with these plants.

**Table 5:** The common phytochemical compounds present in the essential oils of selected *Helichyrsum species*.

*Helichrysum* Species	Plant Parts	Compounds	Method of Analysis	Pharmacological Activity	References
***H. petiolare*** 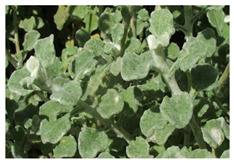 **A growing *Helichrysum petiolare* plant [70] SANBI available online http://pza.sanbi.org/helichrysum-petiolare (accessed on 19 June 2021)**	Leaves	α-pinene (6.8%), 1, 8-cineole (22.4), p-cymene (9.8%) and β-caryophyllene (14%)	G.C.-M.S.	Anti-fungal, anti-inflammatory	[38]
	Whole plant	(*E*)-Longipinane (11.79%), *trans*-Geranylgeraniol (11.68%), Phytol (11.28%) Geranyllinalool (11.13%) and α-Eicosane (12.07%)	G.C.-M.S.	Anti-microbial, anti-inflammatory	[71]
***H. cymosum*** 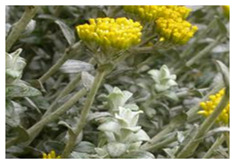 **A growing *Helichrysum cymosum* plant [72] SANBI available online: http://pza.sanbi.org/helichrysum-cymosum-subsp-cymosum (accessed on 19 June 2021)**	Leaves, Flowers	∆-3-carene (16.1%), β-caryophyllene (12.0%)	G.C., G.C.-M.S.	Anti-fungal	[73]
	Flowers	Monoterpenes (77.9%)	G.C.-M.S. G.C., G.C.-M.S.	Anti-inflammatory	[15]
	Leaves, Flowers	(Z) -β- ocimene	G.C.-M.S.	-	[74]
	Leaves	α-pinene (12.4%), 1, 8-cineole (20.4%), β-caryophyllene (10.8%)		Anti-bacterial	[38]
***H. foetidum*** 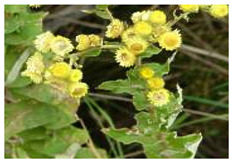 **A growing *Helichrysum foetidum* plant [39] SANBI available online http://pza.sanbi.org/helichrysum-foetidum (accessed on 19 June 2021)**	Leaves, flower	Β-pinene (3.1%), Trans-Sabiene hydrate (1.8%), 4-terpineol (3.1%), β-caryophyllene (2.5%)	G.C.-M.S.	Anti-microbial Anti-inflammatory	[75]
***H. pandurifolium*** 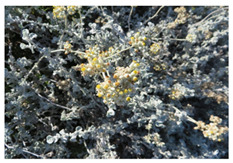 **A growing *Helichrysum pandurifolium* plant [76] iNaturalist. Available online: https://www.inaturalist.org/observations/23571154 (accessed on 19 June 2021)**		N/A	N/A	N/A	N/A

Gas chromatography: G.C.; Gas chromatography mass spectrometry: G.C.-M.S.; Not available: N/A.

## 9. Conclusions

Plants belonging to the *Helichrysum* genus are a vital source of traditional medicines in many parts of the world, including South Africa. The many novel chemical compounds present in the extracts of these plants and their essential oils account for most of their pharmacological actions. This review highlighted the reported diversity of the *Helichrysum* genus and the ethnomedicinal and biological activities of some of its species. However, only limited scientific reports are available in the literature on some species, hence further multi-disciplinary studies by botanists, chemists, ethnopharmacologists and medical scientists are required on all the plants in this genus, as they appear to be potential sources of useful bioactive medicinal compounds that could be exploited in the drug discovery and development value chain.

## Figures and Tables

**Table 1:** Description of bioactivity of the compounds of *Helichrysum cymosum*.

Compounds	Biological Functions
Helihumulone	anti-bacterial and anti-mycotic [15,52]
(Z)-β-ocimene	Molluscidal and leshimanicidal agents [53]
Trans-caryophyllene	Anti-malarial [15,36]
1, 8-cineole	Anti-inflammatory, antioxidant, anticancer, analgesic [54]
α-humulene	Anti-Proliferative [55]
(E)-β-ocimene	Molluscidal and leshimanicidal agents [53]
Caryophyllene oxide	Anti-malarial [15,36]
β-caryophyllene	Anti-malarial [15,36]
Δ-3-carene	AChE inhibition, anti-inflammatory Anti-fungal [56,57]
5-hydroxy-8-methoxy-7-prenyloxyflavanone	Anti-viral [30]

**Table 2:** Description of bioactivity of the compounds *Helichrysum foetidum*.

Compounds	Biological Functions
6′-methoxy-2′, 4, 4′-trihydroxychalcone	Anti-bacterial, anti-fungal, anti-ulcerogenic [43]
6′-methoxy-2′,4-dihydroxychalcone- 4′-O-β-D-glucoside	Anti-bacterial, anti-fungal, anti-ulcerogenic [43]
Kaur-16-en-18-oic acid	Anti-bacterial, anti-fungal, anti-ulcerogenic [43]
Apigenin	Anti-bacterial, anti-fungal, anti-ulcerogenic [43]
Apigenin-7-O-β-D-glucoside	Anti-bacterial, anti-fungal, anti-ulcerogenic [43]
7,4′-dihydroxy-5-methoxy-flavanone	Anti-bacterial, anti-fungal, anti-ulcerogenic [43]
4,5 –diacaffeoyl quinic acid	Anti-bacterial, anti-fungal, anti-ulcerogenic [43]
Ferulic acid	Anti-inflammatory, anti-oxidant, anti-diabetic, anti-hypertensive [58]
Chlorogenic acid	Anti-oxidant, anti-inflammatory, Anti-bacterial, anti-mutagenic and anti-cancer [59]
Campest-7-en-3-ol	Anti-bacterial, antifungal, anti-ulcerogenic [43]

**Table 3:** Description of bioactivity of the compounds of *Helichrysum petiolare*.

Compounds	Biological Functions
Alloaromadendrene	Anti-microbial, anti-viral, anti-diabetic, anti-inflammatory [60]
δ-Selinene	anti-fungal [61]
Phenol, 2-ethoxy-5- (1, 2-dihydroxyethyl) -	N/A
Phytol	Anti-oxidant, anti-microbial, anti-convulsant, cytotoxic, anti-inflammatory [62]
Ledol	N/A
α-Calacorene	Anti-microbial, anti-oxidant [63]
β-Hydroagarofuran	Anti-bacterial and anti-mycotic [15]
Spathulenol	Anti-inflammatory, anti-nociceptive [64,65]
α-Bulnesene	Anti-platelet aggregation agent [66]
δ-Cadinene	Anti-malarial [67]

Not available: N/A.

**Table 4:** Description of bioactivity of the compounds of *H. pandurifolium*.

Compounds	Biological Functions
Viridiflorol	Anti-fungal, anti-bacterial [14]
Pogostol	N/A [14]
α-pinene	N/A [14]
β-pinene	N/A [14]
Aristolochene	N/A [14]
(E)-1(6,10-dimethylundec-5-en-2-yl)-4-methylbenzene	N/A [14]
Z-β-ocimene	N/A [14]
δ-cadinene	N/A [14]
Germacrene d-4-ol	N/A [14]
1,8-cineole	N/A [14]

Not available: N/A.

## Data Availability

Not applicable.
